# Deep learning-based classification of the capillary ultrastructure in human skeletal muscles

**DOI:** 10.3389/fmolb.2024.1363384

**Published:** 2024-05-01

**Authors:** Marius Reto Bigler, Oliver Baum

**Affiliations:** ^1^ Department of Cardiology, Inselspital, Bern University Hospital, University of Bern, Bern, Switzerland; ^2^ Institut für Physiologie, Charité–Universitätsmedizin Berlin, Berlin, Germany

**Keywords:** capillaries, skeletal muscle, transmission electron microscopy, convolutional neural networks, deep learning

## Abstract

**Background:**

Capillary ultrastructure in human skeletal muscles is dynamic and prone to alterations in response to many stimuli, e.g., systemic pathologies such as diabetes mellitus and arterial hypertension. Using transmission electron microscopy (TEM) images, several studies have been conducted to quantify the capillary ultrastructure by means of morphometry. Deep learning techniques like convolutional neural networks (CNNs) are utilized to extract data-driven characteristics and to recognize patterns. Hence, the aim of this study was to train a CNN to identify morphometric patterns that differ between capillaries in muscle biopsies of healthy participants and patients with systemic pathologies for the purpose of hypothesis generation.

**Methods:**

In this retrospective study we used 1810 electron micrographs from human skeletal muscle capillaries derived from 70 study participants which were classified as “healthy” controls or “patients“ in dependence of the absence or presence of a documented history of diabetes mellitus, arterial hypertension or peripheral arterial disease. Using these micrographs, a pre-trained open-access CNN (ResNet101) was trained to discriminate between micrographs of capillaries of the two groups. The CNN with the highest diagnostic accuracies during training were subsequently compared with manual quantitative analysis of the capillary ultrastructure to distinguish between “healthy” controls and patients.

**Results:**

Using classification into controls or patients as allocation reference, receiver-operating-characteristics (ROC)-analysis of manually obtained BM thickness showed the best diagnostic accuracy of all morphometric indicators (area under the ROC-curve (AUC): 0.657 ± 0.050). The best performing CNN demonstrated a diagnostic accuracy of 79% (sensitivity 93%, specificity 92%). DeLong-Test of the ROC-curves showed a significant difference (*p* < 0.001) between the AUC of the best performing CNN and the BM thickness. The underlying morphology responsible for the network prediction focuses mainly on debridement of pericytes.

**Conclusion:**

The hypothesis-generating approach using pretrained CNN distinguishes between capillaries depicted on electron micrographs of “healthy” controls and participants with a systemic pathology more accurately than by commonly used morphometric analysis.

## Graphical Abstract

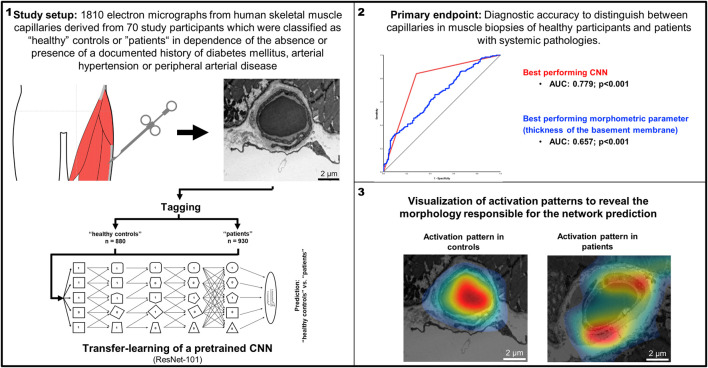

## Introduction

Capillaries are the sections of the vascular system with the most narrow diameter (Tuma et al., 2011). They branch from arterioles to meander through the tissues and then drain into collecting venules and ensuing veins. According to the law of Hagen-Poisseuille, which states that the blood flow velocity is proportional to the fourth power of the vessel radius, this transition from the arterioles into the capillary network is accompanied by a significant reduction in the velocity of the blood flow. Of note, as the transition into smaller vessels results in a significant increase in the overall diameter of the arterial vascular system, the total blood flow remains constant, i.e., the cardiac output. The reduction of the blood flow velocity in the capillary system ensures that the red blood cells release ample oxygen amounts to supply the surrounding tissues during their microcirculation passage and, in addition, facilitates the essentially balanced exchange of energy substrates and metabolic end products between the vascular system and the tissue.

As most clearly visualized using transmission electron microscopy (TEM), capillaries are of simple structure. Endothelial cells (ECs) close together as the vessel wall in such a way that a capillary lumen is formed. The abluminal surface of the ECs is covered by a continuous basement membrane (BM) mainly consisting of collagen type IV and other extracellular matrix (ECM) components such as laminin, heparan-sulphate proteoglycans (HSPGs) and nidogen/entactin (Kalluri, 2003). Pericytes (PC) are embedded in this BM and wrap their protrusions abluminally around the ECs. These contractile cells may influence the capillary blood flow in many tissues and communicate with the underlying ECs to influence the functional integrity of the capillaries (Armulik et al., 2011; Yamazaki and Mukouyama, 2018).

The capillary phenotype is dynamic. Inflation of the ECs volume during ischemia highlights the structural versatility of capillaries (Egginton and Hudlická, 1999). Furthermore, the thickness of the peri-capillary BM in human skeletal muscles increases in common cardiovascular diseases such as peripheral arterial disease (PAD), diabetes mellitus or arterial hypertension (Baum et al., 2020), but decreases in response to physical activity (Williamson et al., 1996). Strikingly, the BM thickening is accompanied by significant changes in the pathophysiology of the capillaries (Baum and Bigler, 2016).

Sophisticated methodological approaches have been developed in recent years that significantly improved the ultrastructural analysis by means of TEM been applied for more than 50 years. However and despite some simplifications (e.g., tablet-based image analysis (TBIA) (Bigler et al., 2016)), the quantitative evaluation of the images is still largely manually performed, posing a challenge for the morphometric processing of large amounts of data. In addition, the morphometry rules stipulate that the morphological features to be assessed are defined in advance, which means that changes in the capillary structure related to the pathophysiology could remain undetected during the analysis due to a selection bias. In contrast, deep learning methods such as convolutional neural networks (CNN) are not affected by this selection bias. Instead, the algorithm tries to find patterns in data sets to solve a pre-defined task without observer guidance (LeCun et al., 2015; Goodfellow et al., 2016).

We hypothesized that a deep learning-based approach with transfer learning of open-available, pre-trained CNN allows the identification of morphometric patterns that differ between capillaries in muscle biopsies of healthy participants and patients with systemic pathologies for the purpose of hypothesis generation. Thus, the aim of the study was to train a CNN and subsequently visualize its activation patterns to demonstrate the triggering morphology for the network prediction. In a second step, we compared the results obtained applying the deep learning-based approach with data based on classic morphometry (i.e., the conventional method).

## Methods

### Study participants and muscle biopsies

For this retrospective study electron micrographs of capillaries were used, that were taken by transmission electron microscopy on biopsies of the vastus lateralis muscle (VL). The biopsies were derived from human participants of five studies conducted at the Department of Anatomy, University of Bern (Rosler et al., 1986; Suter et al., 1995), the University of Copenhagen (Nyberg et al., 2012; Winding et al., 2018), or the University of the sunshine Coast, Australia (Walker et al., 2016). Written informed consent was obtained in each case prior to the study beginning. In all investigations, the criteria and ethical guidelines for treatment of human participants conform to the principles outlined in the Declaration of Helsinki were fulfilled. Each study protocol was approved by the local ethics committee responsible for supervision at the time of study execution, as described earlier (Rosler et al., 1986; Suter et al., 1995; Nyberg et al., 2012; Hoier et al., 2013; Walker et al., 2016; Winding et al., 2018).

The VL muscle biopsies were taken by authorized medical practitioners using Bergstroem needles after local subcutaneous analgesia and immediately fixed in 6.25% (v/v) glutaraldehyde buffered with 0.1 M sodium cacodylate–HCl (pH 7.4) to be stored at 4°C until analysis. Ultrathin sections of the muscle biopsies were prepared and subjected to TEM analysis to record electron micrographs, as previously described in detail (Baum et al., 2020).

For this analysis, participants were classified as “healthy” controls or ‘patients’ in dependence of the absence or presence of a documented history of diabetes mellitus, PAD or arterial hypertension. Application of these criteria resulted in 42 controls and 28 patients, providing a total of 1810 electron micrographs of capillary profiles. In the patient group, 9 patients had a documented history of arterial hypertension, 10 patients had diabetes mellitus and 9 patients had clinically relevant PAD.

### Capillary morphometry

Study parameters were adopted from the original studies, i.e., lumen radius (in nm), thickness of the endothelial cell (in nm), thickness of the BM (in nm) as well as capillary radius (in nm). Furthermore, all study parameters including pericyte cells were calculated as fraction of the capillary area (in %) (Baum and Bigler, 2016).

### General principle of the applied deep-learning based method

The construction and training of a complex CNN architecture requires a large dataset and training over a considerable period of time, even for the establishment of general pattern recognition. To streamline this process, we employed openly accessible pre-trained CNN models that were trained using an extensive dataset from the ImageNet Large Scale Visual Recognition Challenge (Deng, 2009; ILSVRC; http://www.image-net.org/challenges/LSVRC/). Consequently, this approach enables the utilization of a complex network architecture even with a limited dataset. However, a drawback of this method is the pre-defined input layer, which requires data adjustments such as resizing to match the selected networks. In a next step, the output layers of these pretrained CNN are replaced to fit the new task. After the training phase during which the CNN learns to perform the new task, its performance is evaluated using a new dataset. Subsequently, in the final step, the CNNs exhibiting the highest performance are subjected to additional analysis in order to visualize the specific regions within the images that contribute to the CNN’s decision-making process (i.e., the trigger morphology).

### Computational hardware

Network training was simultaneously performed on two computers (Intel^®^ Core™ i7-7700 CPU@3.60GHz, 8GB RAM respectively Intel^®^ Core™ i7-8550U CPU@1.80GHz, 8GB RAM) using customized software (written in Matlab R2019b and R2020a).

### Randomization, image allocation and preparation

**FIGURE 1:**
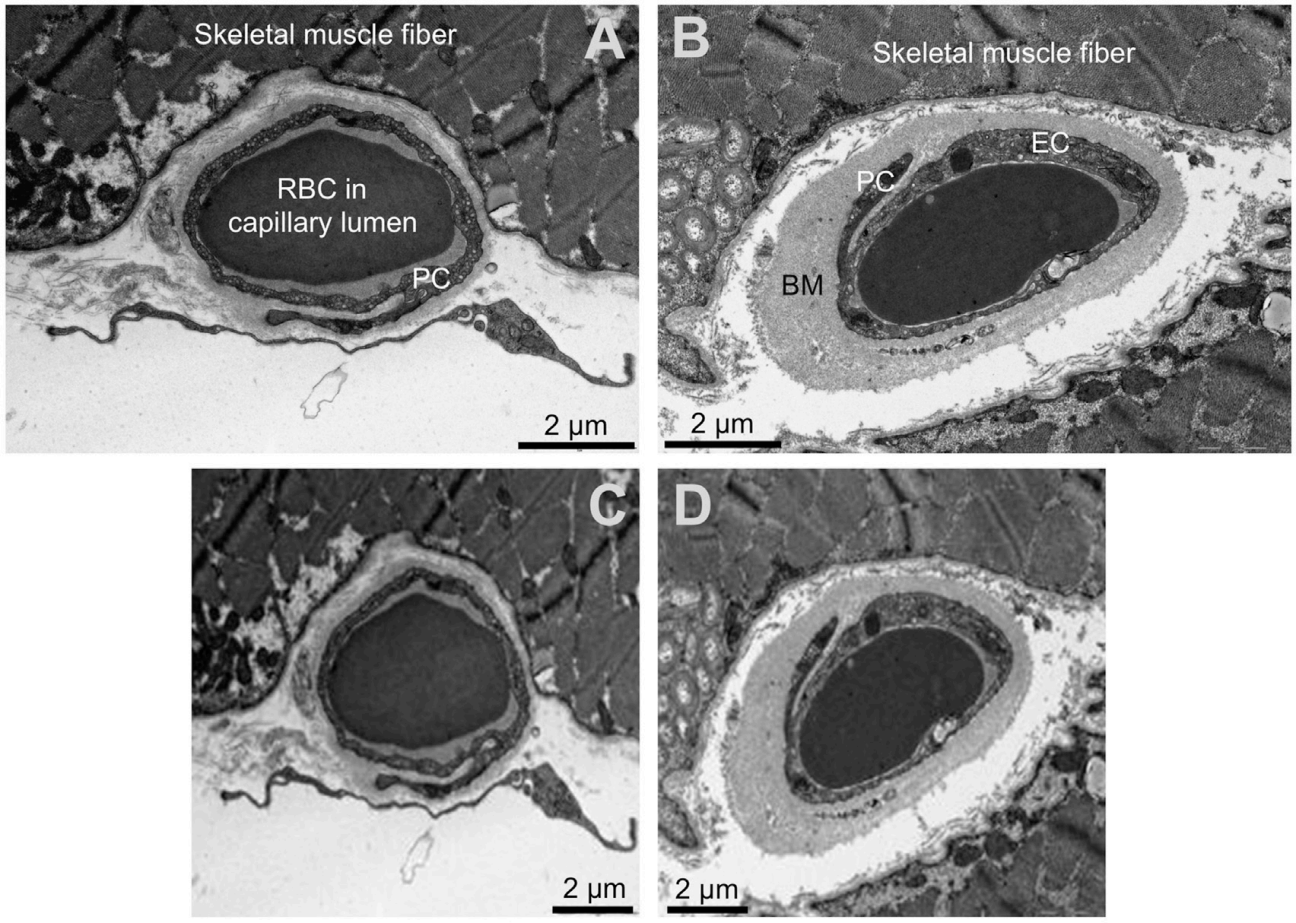
Input data for the neural networks. Upper panels **(A, B)**: Original transmission electron microscopy images of the human skeletal muscle capillaries. **(A)** capillary of a healthy participant, **(B)** capillary of a patient with diabetes mellitus. Lower panels **(C,D)**: Input images for the convolutional neuronal networks after required adjustment of the dimension. Please note the loss of resolution. The scale was not available for the networks and later added for better visualization. RBC = red blood cell PC = pericytes, EC = endothelial cell, BM = basement membrane.

Initially, randomization on participant level intro training, validation and examination data (75% respectively 25% (validation + examination) as recommended by Goodfellow et al. (Goodfellow et al., 2016)) was performed using a random number vector to avoid overfitting of single participants, resulting in 52 participants in the training group, 13 in the validation group and five in the examination group. Electron micrographs (Figure 1 upper panel) were then plotted in Matlab, saved as jpg-images with predefined image size (224 × 224 × 3 pixels, Figure 1 lower panel) and stored in group-specific folders (880 control and 930 pathologic images).

Prior to each training iteration, all training images were randomly shuffled and processed by adding data noise to prevent overfitting (Goodfellow et al., 2016; Trask, 2020). Therefore, the images were randomly rotated in a range between ±45° and translocated ±10 pixels in each direction.

### Selection and preparation of the pretrained convolutional neural networks

For this study, we applied ResNet101, a 101 convolutional layer deep CNN developed by He et al. (He et al., 2015). ResNet101 uses a special residual learning framework allowing the training of a deeper and thus more accurate network compared to other network architectures (i.e., GoogLeNet (Szegedy et al., 2014)) in terms of diagnostic accuracy.

To prepare for the transfer learning process, the last three layers of the networks responsible for the network prediction had to be replaced for the new task, i.e., classification of electron micrographs into either the control or the pathologic group. In addition, a dropout layer was added to prevent the network from overfitting (Srivastava et al., 2014). The remaining layers accounting for pattern recognition and feature extraction were not changed. General learning rate was chosen low while the new layers received a learning rate weight factor of 10 (i.e., 10-fold the normal learning rate) to improve and accelerate their training process.

### Training of the neural networks

Hyperparameter optimization of the transfer learning process was performed for three parameters, i.e., learning rate, dropout probability and minibatch size, using Bayesian optimization technique (Bengio, 2000) and the adaptive moment estimation learning rate algorithm, ADAM (Diederik and Ba, 2014)). Validation of the network performance was performed every ten iterations. Further, a preliminary termination term was added to the algorithm, which terminated the training process when the validation and the training performances diverged twenty times in a row.

### Network performance analysis

Networks performing above the arbitrary threshold of 60% classification accuracy (i.e (true positive + true negative)/(true positive + true negative + false positive + false negative)) on the validation data during the training process were stored for in-depth evaluation with determination of diagnostic accuracy on each subset (i.e., the validation data and the examination data) as well as the combined data sets. Based on the results of this evaluation, the best three networks were further evaluated with class activation mapping (CAM) (Zhou et al., 2015; Selvaraju et al., 2017), i.e., parametric visualization of their activation patterns to find the morphology responsible for the network prediction using ten characteristic electron micrographs (Supplementary Figure S2). When multiple networks showed similar performance, the network with the smallest discrepancy between validation and examination data was selected.

### Statistical analysis

Two study groups (“healthy” and “patients” based on the above mentioned classification in controls or patients in dependence of the absence or presence of a documented history of diabetes mellitus, PAD or arterial hypertension were formed. Between-group comparison of continuous study parameters was performed by an unpaired Student’s t-test. Network performance was analyzed by determination of classification accuracy (i.e., correct classified images/all images) using a 4-field matrix and calculation of sensitivity, specificity and F1-score (harmonic mean of sensitivity and positive predictive value). Nonparametric receiver operating characteristics (ROC) analysis was performed for accuracy assessment of differentiating between electron micrographs of controls or patients by manually obtained study parameters (continuous) and the CNN prediction (dichotomous). Comparison of the area under the ROC curves was performed using the DeLong-Test.

Statistical significance was defined at a p-level of <0.05. Continuous variables are given as mean ± standard deviation. All analyses were performed using SPSS version 25 (IBM Statistics, Armonk, New York) or MedCalc for Windows, version 19.1 (MedCalc Software, Ostend, Belgium).

## Results

1810 electron micrographs from 70 participants were included in the study, among these 880 micrographs were derived from muscle biopsies of 42 healthy control subjects and 930 from those of 28 patients. 1,347 were used for the training and 463 electron micrographs for the performance evaluation of the CNN (Table 1). Most of the participants were male (69%) with a mean age of 49.2 years (range 23–75 years). Of note, participants included in the patient group were significantly older than participants in the control group (57.6 years *versus* 43.9 years, *p* < 0.001).

**TABLE 1:** Study parameters.

	Controls	Patients	*p*-value
Overall, n	880	930	-
Lumen radius (nm)	1,589 ± 421	1.452 ± 460	*p* < 0.001
Thickness of the endothelium (nm)	421 ± 303	481 ± 290	*p* < 0.001
Thickness of the basement membrane (nm)	218 ± 69	308 ± 118	*p* < 0.001
Capillary radius (nm)	2,406 ± 356	2,429 ± 440	*p* = 0.226
Training data	691	656	-
Lumen radius (nm)	1,589 ± 427	1,443 ± 464	*p* < 0.001
Thickness of the endothelium (nm)	423 ± 317	485 ± 294	*p* < 0.001
Thickness of the basement membrane (nm)	215 ± 67	319 ± 123	*p* < 0.001
Capillary radius (nm)	2,400 ± 361	2,442 ± 422	*p* = 0.047
Validation data	159	231	-
Lumen radius (nm)	1,576 ± 407	1,459 ± 459	*p* = 0.012
Thickness of the endothelium (nm)	419 ± 248	456 ± 276	*p* = 0.190
Thickness of the basement membrane (nm)	232 ± 79	287 ± 98	*p* < 0.001
Capillary radius (nm)	2,425 ± 342	2,388 ± 497	*p* = 0.411
Examination data	30	43	-
Lumen radius (nm)	1,672 ± 352	1,558 ± 393	*p* = 0.212
Thickness of the endothelium (nm)	375 ± 228	524 ± 290	*p* = 0.021
Thickness of the basement membrane (nm)	211 ± 50	252 ± 99	*p* = 0.037
Capillary radius (nm)	2,452 ± 304	2,449 ± 377	*p* = 0.971
Validation + Examination data	189	274	-
Lumen radius (nm)	1,591 ± 400	1,477 ± 449	*p* = 0.006
Thickness of the endothelium (nm)	412 ± 245	469 ± 279	*p* = 0.030
Thickness of the basement membrane (nm)	229 ± 75	281 ± 99	*p* < 0.001
Capillary radius (nm)	2,429 ± 336	2,397 ± 480	*p* = 0.428

### Descriptive statistics

**FIGURE 2:**
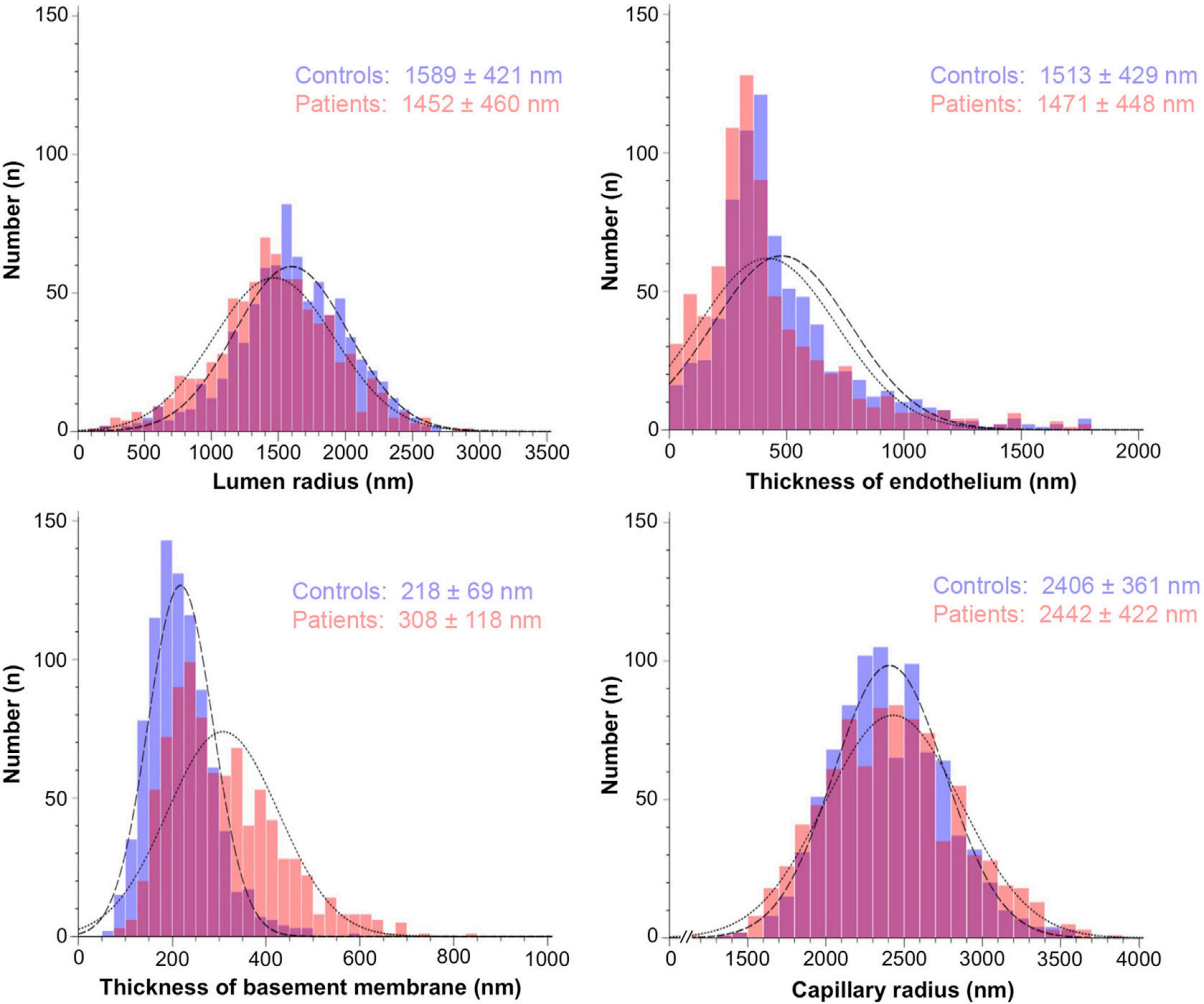
Histograms representing the frequency distribution of the study parameters grouped by the absence or presence of systemic pathologies. The morphometric values for the capillary structure of 880 electron micrographs from control participants (blue bars) and 930 electron micrographs from patients (red bars) were taken from the original studies listed in Materials/Methods. They were determined using tablet-based image analysis and represent mean ± standard deviation.

Descriptive statistics of the study parameters grouped according to the classification “healthy” and “patients” are presented in Table 1; Figure 2 (respectively Supplementary Table S1; Supplementary Figure S1 for the fraction values). Overall, endothelial cell thickness and BM thickness were significantly different between the groups in each data set.

### Receiver-operating characteristic curves

#### Complete data

**FIGURE 3:**
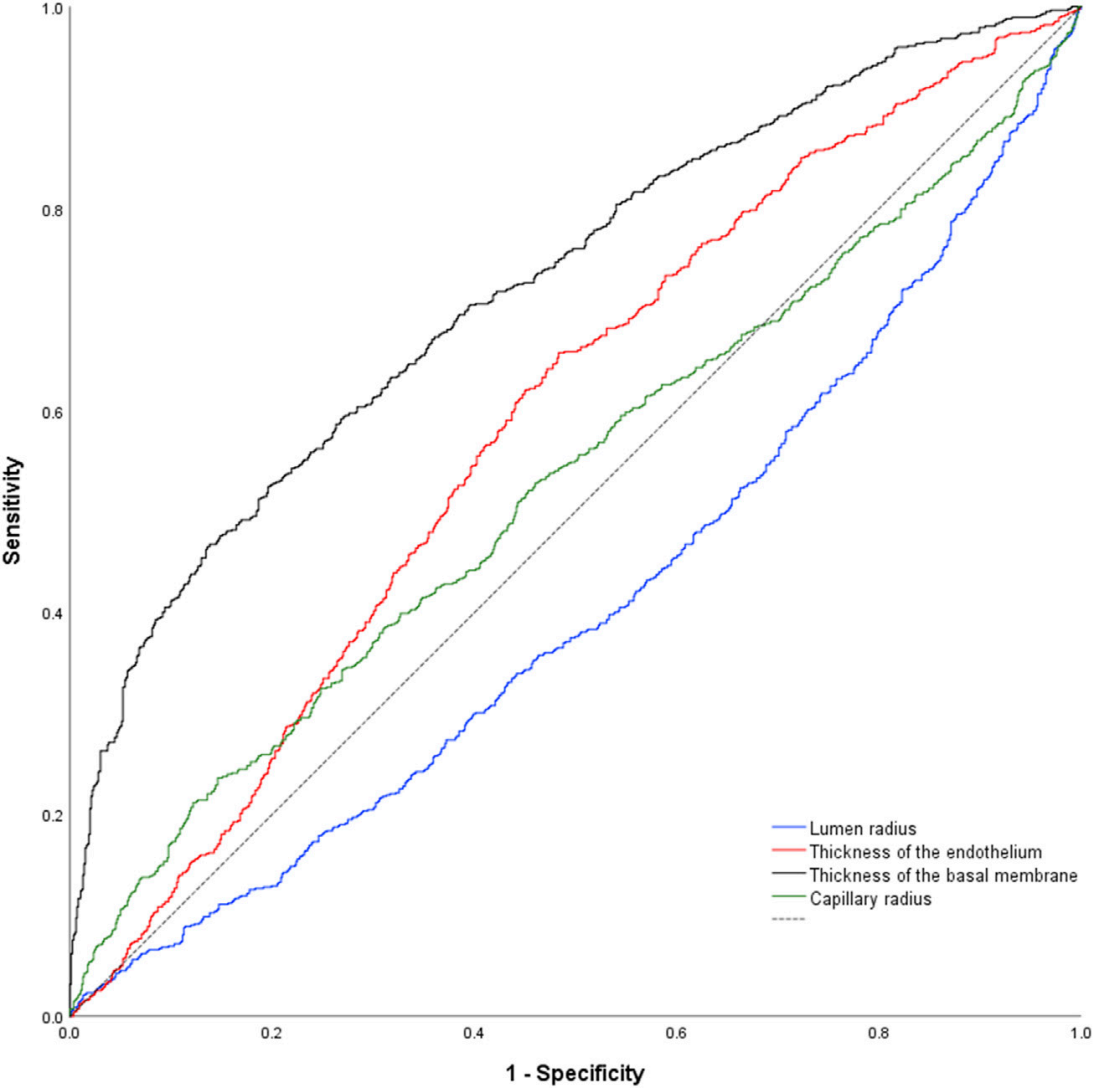
Nonparametric receiver-operating characteristic curve of the study parameters using the complete data set. Of note, all parameters but capillary radius were higher in samples of pathologies. As consequence, the data set for capillary radius is below the reference line (dashed black line).

Using classification into controls or patients as allocation reference, receiver-operating-characteristics (ROC) analysis of the lumen radius showed an area under the ROC-curve of 0.592 ± 0.027 (*p* < 0.001; Figure 3). AUC for endothelial thickness was 0.588 ± 0.027 (*p* < 0.001), for BM thickness 0.743 ± 0.023 (*p* < 0.001) and for the capillary radius 0.511 ± 0.027 (*p* = 0.419).

DeLong-Test of the ROC-curves showed a significant difference of AUC for BM thickness in comparison to all other parameters (*p* ≤ 0.0001). There was no significant difference between the AUCs of the lumen radius and endothelial thickness (*p* = 0.844), but a significant difference of these parameters and capillary radius (*p* = 0.013 respectively *p* ≤ 0.001).

### Validation and examination data

Using classification into controls or patients as allocation reference, ROC analysis of the lumen radius showed an area under the ROC-curve of 0.580 ± 0.054 (*p* = 0.004). AUC for endothelial thickness was 0.574 ± 0.055 (*p* = 0.009), for BM thickness 0.657 ± 0.050 (*p* < 0.001) and for the capillary radius 0.532 ± 0.053 (*p* = 0.235).

Regarding the optimum cut-off of the study parameters, a lumen radius of 1,332 nm distinguished best between control and patient, sensitivity 36%, specificity 80%. The best cut-off point for endothelial thickness was 368 nm (sensitivity 58%, specificity 88%), for BM-thickness 314 nm (sensitivity 32%, specificity 91%) and for capillary radius 2,182 nm (sensitivity 36%, specificity 80%). Of note, lumen and capillary radius decreased with presence of pathologies. Thus, the optimum cut-off points for these parameters were inversely set (i.e., pathologic below 1332nm respectively 2,182 nm). Using these thresholds obtained in the validation and examination data, diagnostic accuracy was calculated to allow a comparison with the CNN (Table 2). Of note, due to missing data for all but BM data, different (lower) diagnostic accuracies are shown than presented in the ROC analysis. Further, absolute numbers of the study parameters are dependent on biopsy fixation and storage and are not generally representative.

**TABLE 2:** Prediction and performance of the study parameters and the networks.

Data	Validation Data				Examination Data				Validation + Examination Data			
Parameter	True	Normal	Pathologic	Accuracy	True	Normal	Pathologic	Accuracy	True	Normal	Pathologic	Accuracy
	Predicted				Predicted				Predicted			
**Lumen radius** Cut-off: ≤1332nm	Normal	122	120	55.90	Normal	27	33	50.68	Normal	149	153	55.01
	Pathologic	37	77		Pathologic	3	10		Pathologic	40	87	
**EC-Thickness** Cut-off: ≥368nm	Normal	86	82	56.64	Normal	22	17	65.75	Normal	108	99	57.98
	Pathologic	72	113		Pathologic	8	26		Pathologic	80	139	
**BM-Thickness** Cut-off: ≥314 nm	Normal	141	155	55.64	Normal	30	31	57.53	Normal	171	186	55.94
	Pathologic	18	76		Pathologic	0	12		Pathologic	18	88	
**Capillary radius** Cut-off: ≤2182 nm	Normal	127	144	54.87	Normal	24	32	47.95	Normal	151	176	53.78
	Pathologic	32	87		Pathologic	6	11		Pathologic	38	98	
**ResNet1:**L4.4e-5_D0.69_M8	Normal	106	22	80.77	Normal	30	22	69.86	Normal	136	44	79.05
	Pathologic	53	209		Pathologic	0	21		Pathologic	53	230	
**ResNet2:**L4.8e-5_D0.32_M13	Normal	92	16	78.72	Normal	26	21	65.75	Normal	118	37	76.67
	Pathologic	67	215		Pathologic	4	22		Pathologic	71	237	
**ResNet5:**L1e-4DO51M31	Normal	82	13	76.92	Normal	25	19	67.12	Normal	107	32	75.38
	Pathologic	77	218		Pathologic	5	24		Pathologic	82	242	

Order according to accuracy. L = learning rate, D = dropout rate, M = minibatch size.

Of note, there were missing data for lumen radius, EC-Thickness and Capillary radius resulting in a smaller total number of cases.

### Study parameter and network performance

**FIGURE 4:**
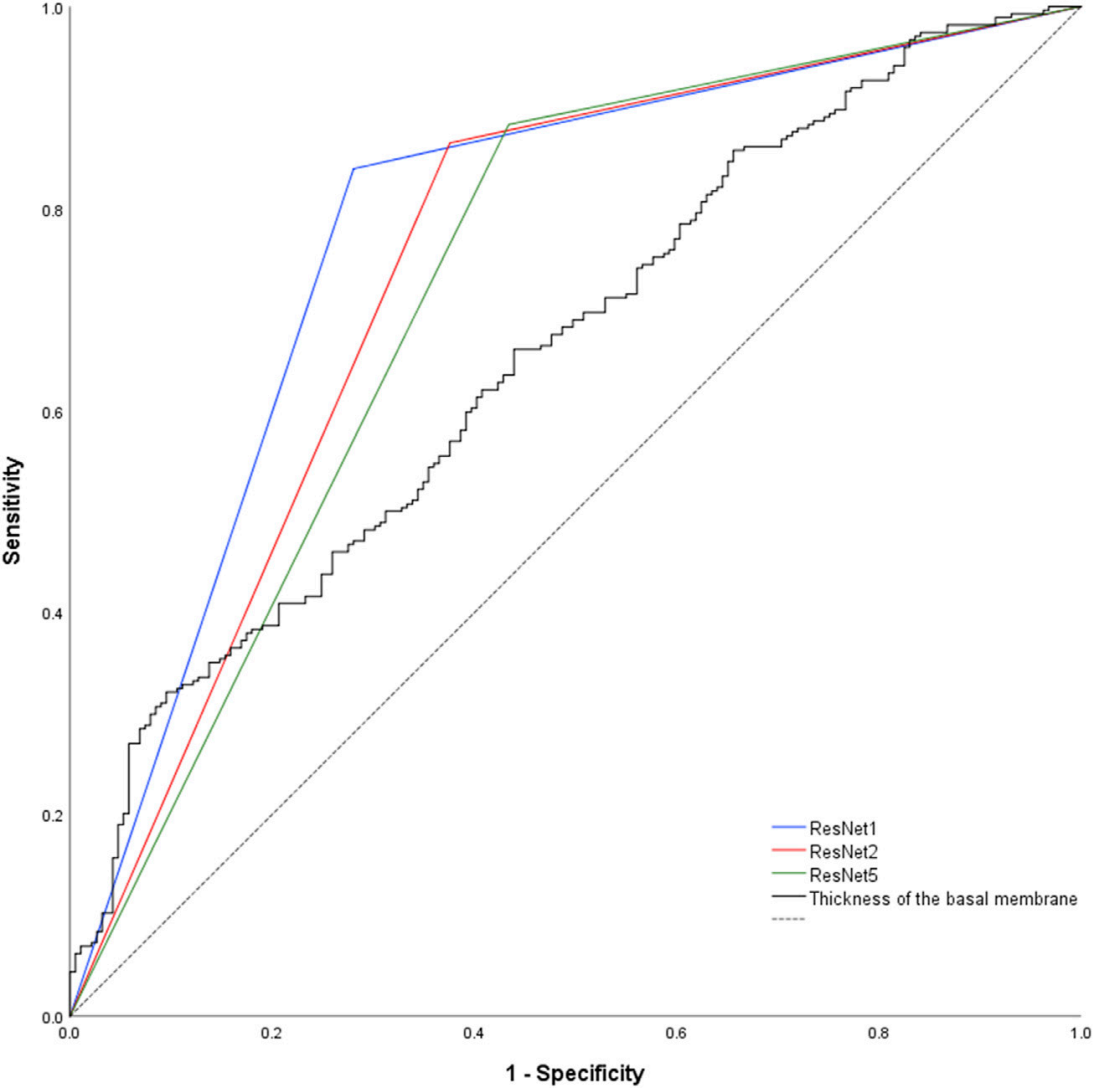
Nonparametric receiver-operating characteristic curve of the basement membrane thickness and the network predictions using the validation and examination data. Of note, network prediction provides a dichotomous output (“healthy control” respectively “patient”), resulting in a triangular ROC-curve. Hence, there is only one combination of sensitivity and specificity possible for each CNN. Dashed black line = reference line.

**FIGURE 5:**
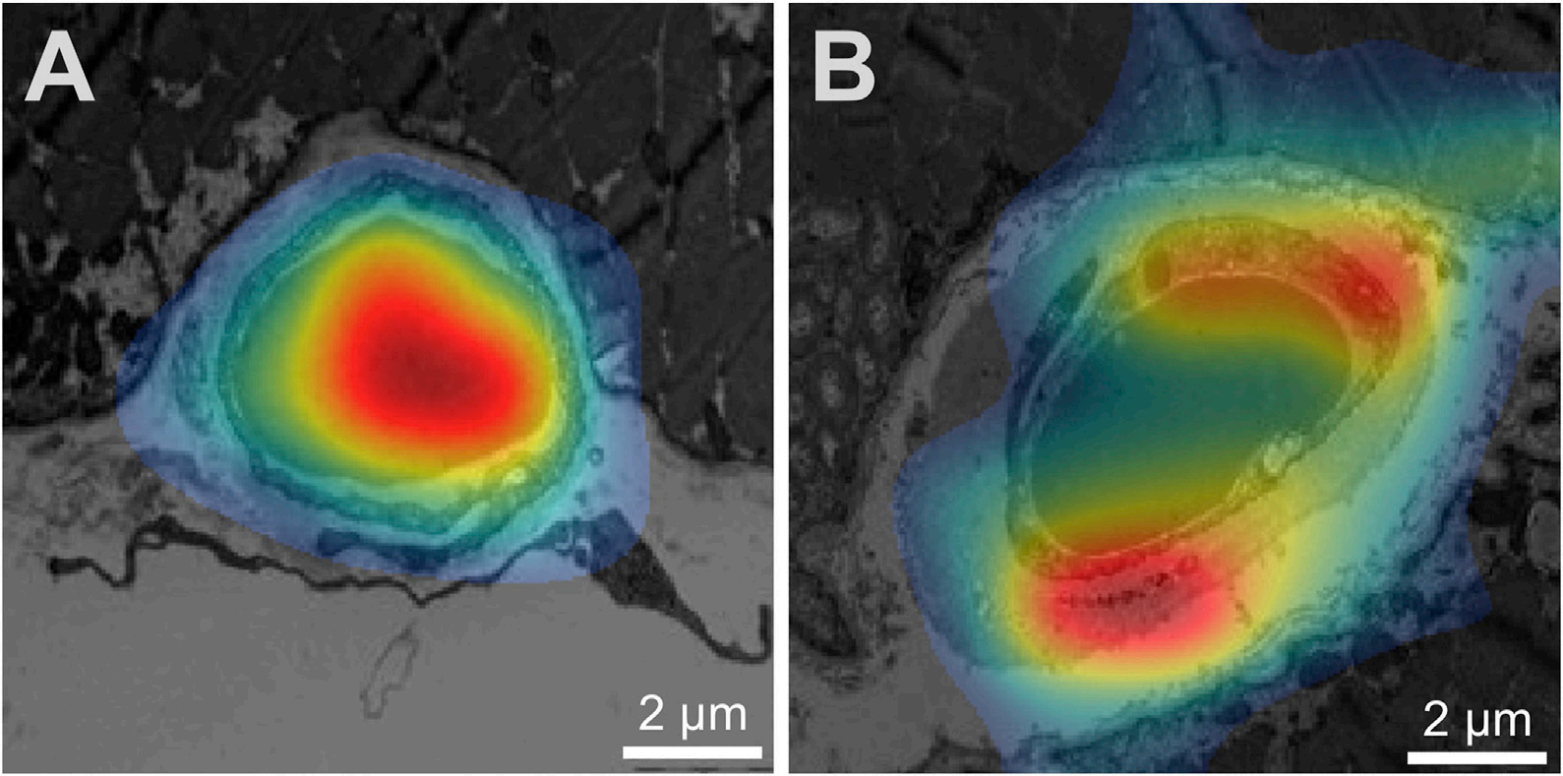
Visualization of network activation patterns of the best performing CNN **(A)** Capillary of a healthy participant, **(B)** capillary of a patient. Red regions contributed most to the network class prediction. Hence, the electron micrograph of the patient was recognized by the debridement of the pericyte (lower red region) and the thickness of the endothelium cell (upper red region). Of note, the scale was later added for better visualization.

Prediction of the study parameters and the three best performing networks including their accuracy, sensitivity and specificity are presented in Table 2. Using participant allocation as reference for the ROC analysis, the three best-performing networks showed a diagnostic accuracy of 79% (RN1: sensitivity 93%, specificity 92%, AUC 0.779 ± 0.023), 77% (RN2: sensitivity 88%, specificity 96, AUC 0.745 + 0.024) and 75% (RN5: sensitivity 89%, specificity 95%, AUC 0.725 + 0.025; Figure 4). By visualization of the activation patterns on ten characteristic electron micrographs, it could be shown that the underlying morphology responsible for the network prediction focuses primarily on debridement of pericytes and to a lesser extent on the structure of the endothelium. These network activation patterns are depicted in Figure 5, and in detail in Supplementary Figure S2.

### Comparison of study parameter and network performance

Based on the performance of the different morphologic parameters as well as missing data for lumen radius, endothelial thickness and capillary radius, only a comparison with BM thickness was performed.

DeLong-Test of the ROC-curves (Figure 4) showed significant difference of AUCs between BM thickness and the three networks (RN1: *p* < 0.001; RN2: *p* = 0.002; RN5: *p* = 0.015). Further, there was a significant difference between the AUCs of RN1 and RN5 (*p* = 0.003).

## Discussion

In the present project, we used a deep learning-based approach with transfer learning of open-available pre-trained CNN to identify morphometric patterns that differ between capillaries in skeletal muscle biopsies of healthy participants and patients with systemic pathologies. Our most relevant findings were: 1. Electron micrographs of skeletal muscle capillaries from healthy controls and participants with a systemic pathology are more accurately distinguishable by CNN than by commonly used morphometric analysis. 2. The underlying morphology responsible for the network prediction focuses primarily on debridement of pericytes and to a lesser extent on the structure of the endothelium.

### Classification of electron micrographs by established morphometric parameters

Ever since the initial observation that certain pathologies are associated with morphological changes of the peri-capillary BM in skeletal muscles, there have been fundamental discussions regarding the methodology for the quantitative determination of this entity. Originally, the scientific debate was driven by methodological approaches developed by two research groups. Siperstein et al. (Siperstein et al., 1968) determined the capillary basement membrane thickness (CBMT) by calculation the mean of 20 measurements of the distances between the abluminal EC surface and the endomysium that do not intersect a PC profile (”20-line measurement”). On the other hand, Williamson et al. (Williamson et al., 1969) preferred the measurement of the CBMT at the two sites of the capillary profile where the BM appeared smallest (“two-minimum-point technique”). However, both morphometric methods are characterized by a time-intensive nature and exhibit specific technical limitations as previously discussed (Baum and Bigler, 2016). Therefore, given the technological advancements, a novel tablet-based image analysis (TBIA) methodology was developed to facilitate the precise quantitative assessment of CBMT (Bigler et al., 2016). Application of this approach allowed not only accurate and reproducible analysis of the CBMT, but also the assessment of numerous other structural indicators simultaneously during the same analysis. As a result, our study group could not only confirm the direct correlation between hypertension, diabetes mellitus, PAD or age with CBMT (Bigler et al., 2016), but also corroborate the favorable impact of physical exercise on CBMT with a partial reduction (Baum and Bigler, 2016).

### Application of deep-learning on morphometric data/electron micrographs

An increasing number of research groups have applied deep-learning based methodologies in basic science. There, its application has spanned a wide spectrum, encompassing the identification of gold nanoparticles in TEM images of tumor cells (Kaphle et al., 2023), deep-learning assisted segmentation of atomic structures (Sadre et al., 2021), and translational research involving the correlation of deep learning-based kidney histomorphometry with patient data (Ginley et al., 2023). The wide array of applications underscores the versatility of this approach. However, to the best of our knowledge, this study presents the first application of a pretrained CNN-approach on TEM-images of the capillary ultrastructure in human skeletal muscles.

### Comparison of CNN and established morphometric parameters

The primary finding of this study is that transfer learning of a pretrained CNN is accurate for allocating electron micrographs of human skeletal muscle capillaries to healthy controls or participants with a systemic pathology. Noteworthy, its diagnostic accuracy for this allocation is higher than the methods previously used and established morphometric indicators for the evaluation of capillary ultrastructure. Using parametric visualization of the activation patterns, we could demonstrate that CNN focuses on distinctive features of the capillary ultrastructure, in particular debridement of pericytes.

Our findings are in agreement with the current hypothesis on the etiology of capillary BM thickening according to Tilton et al. (Tilton et al., 1981) and Vracko et al. (Vracko and Benditt, 1970). Based on their observation of widespread cellular debris within the thickened BM, they independently proposed a disturbed and incomplete turnover of cells associated with the capillaries including apoptosis and replacement of the degenerated cells by new pericyte precursor cells, which then differentiate and generate a new BM layer. Consequently, the inadequately regulated turnover of PCs results in an accumulation of BM material during each cycle. Of note, this hypothesis would provide an explanation for the frequently observed lamellar structure of the BM in capillary profiles of diabetic patients (Baum and Bigler, 2016), akin to growth rings of trees.

Despite this established hypothesis, a comprehensive quantitative assessment of cellular debris and its correlation with BM thickness have yet to be conducted. Hence and in light of the recent reaffirmation of this pathophysiological explanation by the present study, further quantitative analysis with focus on this phenomenon are required.

## Limitations

The present study has several limitations. First, the categorization of the study participants into the distinct groups “healthy” and “patients” introduced heterogeneity into the data. Based on this dichotomy with its potential complexities, it is conceivable that there exists the potential for undetected arterial hypertension or incipient diabetes in the former group with already initiated microvascular changes. Conversely, pathological cases exhibiting optimal medical management may result in minimal pathophysiological alternations. In summary, variability could lead to a considerable degree of overlap between the groups. Nevertheless, due to the retrospective nature of the study design, the adjustment for these factors was not feasible.

Second, age was the only variable not accounted for by the study design excluding relevant co-morbidities during enrollment in the original studies. However, in a preliminary study by our research team, we could demonstrate that most markers of capillary ultrastructure exhibit only non-significant changes (*p* > 0.05) with age, except for the basement membrane thickness. This exception was attributed rather to an increase in age-related comorbidities (such as hypertension and diabetes), than to the aging process itself (Bigler et al., 2016).

Third, the utilization of transfer learning of a pretrained CNN facilitated the implementation of networks with a high capacity for small data. However, this advantage came at the cost of predefined inputs. In the presented study, this constraint led to a notable reduction in input dimensions and consequently, image resolution. As a result, it is conceivable that nuanced morphological patterns may have escaped detection by the network.

Fourth, the applied strategy with transfer-learning of a pretrained CNN without further adjustments to the output is insufficient for the development of a diagnostic model. Therefore, analysis of the probabilities rather than the binary output, inclusion of the morphometric features as covariates as well as cross-validation would be required to gain prediction stability. Nonetheless, given that the aim of our study is to generate hypotheses that necessitate subsequent validation, the study design offers significant benefits in terms of its simplicity and ease of application.

Last, application of CNN results in a “complicated interconnected hierarchical representations of the training data to produce its predictions” (Lundervold and Lundervold, 2019). Thus, interpretation of these predictions remains intricate, even with the assistance of class activation maps, which provide insight into the general distinction procedure. In this study, the CNN exhibited an astonishing diagnostic accuracy, surpassing that of conventional morphometric parameters. Notwithstanding these results, the depicted activation maps demonstrated a diffuse activation pattern leading to indistinct predictions, which complicates the interpretation even further. However, the CNN’s performance remained consistent across various datasets and, importantly, the results substantiate a biologically plausible underlying pathophysiological mechanism.

## Implications

In this study, the morphometric patterns employed by the CNN for distinguishing between TEM images of capillaries in muscle biopsies from healthy participants and patients with systemic pathologies were innovative, yet rooted in a plausible pathophysiological mechanism. This underscores the feasibility of a hypothesis-generating process using transfer learning of pretrained CNN on a small data set employing single CPU computers. Of note, this approach does not replace the conventional scientific method and further studies, i.e., the quantitative analysis of pericyte debridements across different pathologies, are required to validate the presented findings. However, the study highlights the feasibility of the proposed approach, making it applicable to a diverse range of scientific problems.

## Conclusion

The presented hypothesis-generating approach using pretrained CNN distinguishes electron micrographs of healthy controls and participants with a systemic pathology more accurately than established morphometric analysis. Of note, in addressing this task, the CNN primarily concentrates on debridements of pericytes and thus, a biological plausible mechanism. Hence, demonstrating the feasibility of the hypothesis-generating approach in pretrained CNN on a small data set. However, further quantitative and prospective analyses are required to validate these findings.

## Data Availability

The raw data supporting the conclusion of this article will be made available by the authors, without undue reservation.
