# Supplementary material for: Deep learning-based classification of the capillary ultrastructure in human skeletal muscles
Source: Front Mol Biosci. 2024 May 1;11:1363384. doi: 10.3389/fmolb.2024.1363384 (PMC11094256; doi:10.3389/fmolb.2024.1363384)
Supplement: Supplementary file 1 [file Table1.docx]

# Supplemental tables

Supplemental table 1: Study parameters as fraction of the capillary area

|  | **Controls** | **Patients** | **p-value** |
| --- | --- | --- | --- |
| **Overall, n** | **879** | **836** | **-** |
| Lumen (%) | 47.7±17.0 | 37.4±14.8 | p<0.001 |
| Endothelial cell (%) | 25.3±15.5 | 27.3±13.5 | p=0.004 |
| Basement membrane (%) | 21.2±6.4 | 28.6±9.0 | p<0.001 |
| Pericyte (%) | 6.6±4.2 | 7.1±4.3 | p=0.010 |
| **Training data** | **690** | **595** | **-** |
| Lumen (%) | 47.8±17.2 | 36.6±15.0 | p<0.001 |
| Endothelial cell (%) | 25.4±15.8 | 27.4±13.9 | p=0.018 |
| Basement membrane (%) | 21.0±6.2 | 29.3±9.3 | p<0.001 |
| Pericyte (%) | 6.6±4.2 | 7.0±4.4 | p=0.096 |
| **Validation data** | **159** | **198** | **-** |
| Lumen (%) | 46.1±16.0 | 39.0±13.7 | p=0.012 |
| Endothelial cell (%) | 25.3±14.0 | 26.7±12.6 | p=0.343 |
| Basement membrane (%) | 22.4±7.0 | 27.3±7.9 | p<0.001 |
| Pericyte (%) | 6.6±4.0 | 7.3±4.3 | p=0.094 |
| **Examination data** | **30** | **43** | **-** |
| Lumen (%) | 54.2±16.0 | 40.0±15.6 | p<0.001 |
| Endothelial cell (%) | 22.5±14.9 | 29.2±11.5 | p=0.036 |
| Basement membrane (%) | 19.4±4.9 | 24.1±7.7 | p=0.004 |
| Pericyte (%) | 4.7±3.7 | 6.7±3.2 | p=0.016 |
| **Validation + Examination data** | **189** | **241** | **-** |
| Lumen (%) | 47.4±16.3 | 39.2±14.0 | p<0.001 |
| Endothelial cell (%) | 24.9±14.1 | 27.1±12.4 | p=0.084 |
| Basement membrane (%) | 21.9±6.8 | 26.7±8.0 | p<0.001 |
| Pericyte (%) | 6.3±4.0 | 7.2±4.1 | p=0.019 |
