# Supplementary material for: Deep learning-based classification of the capillary ultrastructure in human skeletal muscles
Source: Front Mol Biosci. 2024 May 1;11:1363384. doi: 10.3389/fmolb.2024.1363384 (PMC11094256; doi:10.3389/fmolb.2024.1363384)
Supplement: Supplementary file 2 [file DataSheet1.DOCX]

# Supplementary data - code

# Image allocation and augmentation

% This is a MATLAB m-file

% --------------------------------------------------------------------

% File : Imagerandomisation_and_allocation.m

% Author : Marius Reto Bigler, adapted from an example from Mathworks

% Institute : Department of Cardiology

% --------------------------------------------------------------------

% Description:

%

% This function randomly allocates all available data into a virtual training and a validation datastore necessary for the transfer learning process.

% Further, it uses data augmentation to prevent overfitting.

% The code is based from an MathWork example and adapted to this task.

parentDir = 'C:\xxx';

dataDirTrain = 'Training';

% create a data store of all training images. Of note, the capillary % TEM images in data 2 are already sorted to the groups "control" and % "patients" according to their medical history

imgsTrain = imageDatastore(fullfile(parentDir,dataDirTrain),...

'IncludeSubfolders',true,...

'LabelSource','foldernames');

dataDirVal = 'Validation';

% then create a data store of all validation images.

imgsValidation = imageDatastore(fullfile(parentDir,dataDirVal),...

'IncludeSubfolders',true,...

'LabelSource','foldernames');

% as well as the examination images

imgsExam = imageDatastore(fullfile(parentDir,'Examination'),...

'IncludeSubfolders',true,...

'LabelSource','foldernames');

% display the number of image in each group

disp(['Number of training images: ',num2str(numel(imgsTrain.Files))]);

disp(['Number of validation images: ',num2str(numel(imgsValidation.Files))]);

disp(['Number of examination images: ',num2str(numel(imgsExam.Files))]);

% define the inputSize

inputSize = [224,224,3];

%% Data augmentation

% Data augmentation (i.e. a augemented data store), transformes batches % of training sets with optional preprocessing such as resizing, % rotation etc.

% This helps to prevent the network from overfitting and memorizing the

% exact details of the training image => in particular recommended with

% limited data.

% define the preprocessing, here with a random rotation as well as with % a shift in both directions (xxx pixels)

imageAugmenter = imageDataAugmenter('RandRotation',[-45,45],...

'RandXTranslation',[-10,10],...

'RandYTranslation',[-10,10]);

% create the augmented data store

augimgsTrain = augmentedImageDatastore(inputSize,imgsTrain,...

'DataAugmentation',imageAugmenter);

%% Variables for BayesianOptimization

XTrain = imgsTrain;

YTrain = imgsTrain.Labels;

XValidation = imgsValidation;

YValidation = imgsValidation.Labels;

# Transfer learning using Bayesian optimization

% This is a MATLAB m-file

% -------------------------------------------------------------------------

% File : deeplearningusingbayesianoptimization.m

% Author : Marius Reto Bigler, adapted from an example from

% Mathworks

% Date adapted : 17.03.2021

% Institute : Department of Cardiology

% -------------------------------------------------------------------------

% Description:

%

% Hyperparameter optimization using Bayesian optimization. After few

% training runs with randomly chosen hyperparameter settings within the

% predefined range, a surrogate function of the cost-function is

% constructed and further hyperparameter settings are tried according to

% the presumed minima.

%

% Please note, explanations are directly copied from the Mathworks example:

% <https://ch.mathworks.com/help/deeplearning/ug/deep-learning-using-bayesian-optimization.html>

%% Deep Learning Using Bayesian Optimization

%

% Choose hyperparameter to optimize

optVars = [

optimizableVariable('InitialLearnRate',[1e-5 2e-4],'Type','real', 'Transform','log')

optimizableVariable('MiniBatchSize',[8 32],'Type','integer', 'Transform','none')

optimizableVariable('Dropout',[0.3 0.8],'Type','real', 'Transform','none')];

% Create the objective function for the Bayesian optimizer, using the

% training and validation data as inputs. The objective function trains a

% convolutional neural network and returns the classification error on the

% validation set. This function is defined at the end of this script.

%

% Because bayesopt uses the error rate on the validation set to choose the

% best model, it is possible that the final network overfits on the

% validation set. The final chosen model is then tested on the independent

% test set to estimate the generalization error.

ObjFcn = makeObjFcn(XTrain,YTrain,XValidation,YValidation);

% Perform Bayesian optimization by minimizing the classification error on

% the validation set. After each network finishes training, bayesopt

% prints the results to the command window.

BayesObject = bayesopt(ObjFcn,optVars);

%% Evaluation

% Load the best network found in the optimization and its validation accuracy.

bestIdx = BayesObject.IndexOfMinimumTrace(end);

fileName = BayesObject.UserDataTrace{bestIdx};

savedStruct = load(fileName);

valError = savedStruct.valError

% Predict the labels of the test set

[YPredicted,probs] = classify(savedStruct.trainedNet,imgsExam);

% Compare it to the true values (i.e., the image labels). Further,

% calculate the test error.

testError = 1 - mean(YPredicted == imgsExam.Labels)

% Plot the confusion matrix for the test data. Display the precision and

% recall for each class by using column and row summaries.

figure('Units','normalized','Position',[0.2 0.2 0.4 0.4]);

cm = confusionchart(imgsExam.Labels,YPredicted);

cm.Title = 'Confusion Matrix for Test Data';

cm.ColumnSummary = 'column-normalized';

cm.RowSummary = 'row-normalized';

%% Objective Function for Optimization

% Define the objective function for optimization. This function performs

% the following steps:

%

% 1) Takes the values of the optimization variables as inputs. bayesopt

% calls the objective function with the current values of the optimization

% variables in a table with each column name equal to the variable name.

%

% 2. Defines the network architecture and training options.

%

% 3. Trains and validates the network.

%

% 4. Saves the trained network, the validation error, and the training

% options to disk.

%

% 5. Returns the validation error and the file name of the saved network.

function ObjFcn = makeObjFcn(XTrain,YTrain,XValidation,YValidation)

ObjFcn = @valErrorFun;

function [valError,cons,fileName] = valErrorFun(optVars)

%% load the pretrained neural network

net = resnet101;

% display the structure of the network including some information

numClasses = numel(categories(YTrain));

lgraph = layerGraph(net);

% remove the previous output layers

lgraph = removeLayers(lgraph,{'fc1000','prob','ClassificationLayer_predictions'});

% define new layers for this specific task including a new classification

% layer with “control” and “patient” classes

newLayers = [dropoutLayer(optVars.Dropout,'Name','newDropout') fullyConnectedLayer(numClasses,'Name','new_fc','WeightLearnRateFactor',10,'BiasLearnRateFactor',10)

softmaxLayer('Name','softmax')

classificationLayer('Name','new_classoutput')];

% add the layers at the end of the CNN and connect them

lgraph = addLayers(lgraph,newLayers);

lgraph = connectLayers(lgraph,'pool5','newDropout');

inputSize = net.Layers(1).InputSize;

numberOfLayers = numel(lgraph.Layers);

% To check that the new layers are connected correctly, plot the new layer

% graph and zoom in on the last layers of the network. (Please note, this

% was only done once and not for each network)

figure('Units','normalized','Position',[0.3 0.3 0.4 0.4]);

plot(lgraph)

ylim([0,10])

% define the training options for the transfer learning. Please note, this

% example was coded with ValidationPatience = “number of times that the

% loss on the validation set can be larger than or equal to the previously % smallest loss before network training stops.” Thus, number of epochs was

% chosen large as the training stopped automatically.

options = trainingOptions('adam',...

'MiniBatchSize',optVars.MiniBatchSize,...

'MaxEpochs',60,...

'InitialLearnRate',optVars.InitialLearnRate,...

'ValidationData',XValidation,...

'ValidationFrequency',10,...

'ValidationPatience',15,...

'Verbose',1,...

'Shuffle','every-epoch',...

'ExecutionEnvironment','auto',...

'Plots','training-progress');

% start with the training process

trainedNet = trainNetwork(datasource,lgraph,options);

% Evaluate the trained network on the validation set, calculate the

% predicted image labels, and calculate the error rate on the validation

% data.

YPredicted = classify(trainedNet,XValidation);

valError = 1 - mean(YPredicted == YValidation);

% save the network using the calculated error rate as name

fileName = num2str(valError) + ".mat";

save(fileName,'trainedNet','valError','options')

cons = [];

end

end

# Network performance analysis

% This is a MATLAB m-file

% -------------------------------------------------------------------------

% File : NetworkPerformanceAnalysis.m

% Author : Marius Reto Bigler, adapted from an example from

% Mathworks

% Date adapted : 19.03.2021

% Institute : Department of Cardiology

% -------------------------------------------------------------------------

% Description:

%

% This function was used to assess the performance of the trained neural

% network. First, it loads the images into a data store and allocates them % into the training group and the validation group. Further, it loads the % examination data from a separate folder. Parts of the code are directly copied from <https://ch.mathworks.com/help/deeplearning/ug/deep-learning-using-bayesian-optimization.html>

% File path

parentDir = 'C:\xxx';

% create a data store of all validation images. Of note, the capillary TEM % images in data 2 are already sorted to the groups "control" and % "patients" according to their medical history

imgsValidation = imageDatastore(fullfile(parentDir,'Validation'),...

'IncludeSubfolders',true,...

'LabelSource','foldernames');

% as well as the examination images

imgsExam = imageDatastore(fullfile(parentDir,'Examination'),...

'IncludeSubfolders',true,...

'LabelSource','foldernames');

%% Predictions

% Predict the labels of the Validation Data Set.

[YPredicted1,probs] = classify(net,imgsValidation);

% Compare it with the true labels and calculate the accuracy.

ValAccuracy = mean(YPredicted1 == imgsValidation.Labels)

% Visualize the results in a confusion matrix

figure(1);

cm1 = confusionchart(imgsValidation.Labels,YPredicted1);

cm1.Title = 'Confusion Matrix for Validation Data';

cm1.ColumnSummary = 'column-normalized';

cm1.RowSummary = 'row-normalized';

% Predict the labels of the Examination Data Set.

[YPredicted,probs] = classify(net,imgsExam);

% Compare it with the true labels and calculate the accuracy.

ExamAccuracy = mean(YPredicted == imgsExam.Labels)

% Visualize the results in a confusion matrix

figure(2);

cm = confusionchart(imgsExam.Labels,YPredicted);

cm.Title = 'Confusion Matrix for Examination Data';

cm.ColumnSummary = 'column-normalized';

cm.RowSummary = 'row-normalized';

# Visualization of network predictions

% This is a MATLAB m-file

% -------------------------------------------------------------------------

% File : InvestigateNetworkPredictions.m

% Author : Marius Reto Bigler, adapted from an example from

% Mathworks

% Date adapted : 19.03.2021

% Institute : Department of Cardiology

% -------------------------------------------------------------------------

% Description:

%

% This function gives us a visual explanation what triggered the prediction

% of the neural network based on the code from the Mathworks example on

% Investigate Network Predictions Using Class Activation Mapping

% (<https://ch.mathworks.com/help/deeplearning/ug/investigate-network-predictions-using-class-activation-mapping.html> )

%

% The class activation map shows which regions of the input image % contributed the most to the predicted class (red = the most).

% Load a pretrained convolutional neural network for image classification.

load('C:\xxx')

% Provide the general network architecture (necessary to know the name of

% the correct output layer required for the class activation mapping

netName = "resnet101";

% Extract the image input size and the output classes of the network.

% The activationLayerName helper function, defined at the end of this

% example, returns the name of the layer to extract the activations from.

% This layer is the ReLU layer that follows the last convolutional layer of

% the network.

inputSize = net.Layers(1).InputSize(1:2);

classes = net.Layers(end).Classes;

layerName = activationLayerName(netName);

%% Display Class Activation Maps

% Create a figure and perform class activation mapping in a loop.

% To terminate execution of the loop, close the figure.

h = figure('Units','normalized','Position',[0.05 0.05 0.9 0.8],'Visible','on');

while ishandle(h)

% File paths of the ten images used to discover the underlying morphology

% responsible for the network prediction.

im = imread('C:\xxx');

% The class activation map for a specific class is the activation map of

% the ReLU layer that follows the final convolutional layer, weighted by

% how much each activation contributes to the final score of that class.

%

% Those weights equal the weights of the final fully connected layer of the

% network for that class. Please note, you can generate a class activation

% map for any output class. For example, if the network makes an incorrect % classification, you can compare the class activation maps for the true

% and predicted classes.

%

% However, here, we are interested in the class activation map for the

% predicted class with the highest score.

scores = squeeze(mean(imageActivations,[1 2]));

if netName ~= "squeezenet"

fcWeights = net.Layers(end-2).Weights;

fcBias = net.Layers(end-2).Bias;

scores = fcWeights*scores + fcBias;

[~,classIds] = maxk(scores,3);

weightVector = shiftdim(fcWeights(classIds(1),:),-1);

classActivationMap = sum(imageActivations.*weightVector,3);

else

[~,classIds] = maxk(scores,3);

classActivationMap = imageActivations(:,:,classIds(1));

end

% Calculate the top class labels and the final normalized class scores.

scores = exp(scores)/sum(exp(scores));

maxScores = scores(classIds);

labels = classes(classIds);

% Plot the class activation map. Display the original image in the first

% subplot. In the second subplot, use the CAMshow helper function, defined

% at the end of this example, to display the class activation map on top of

% a darkened grayscale version of the original image. Display the labels with % their predicted scores.

subplot(1,2,1)

imshow(im)

subplot(1,2,2)

CAMshow(im,classActivationMap)

title(string(labels) + ", " + string(maxScores));

drawnow

end

%% ActivationLayerName function

% provides the correct layer name, i.e., the name of the ReLU layer that

% follows the final convolutional layer

function layerName = activationLayerName(netName)

if netName == "squeezenet"

layerName = 'relu_conv10';

elseif netName == "googlenet"

layerName = 'inception_5b-output';

elseif netName == "resnet18"

layerName = 'res5b_relu';

elseif netName == "resnet101"

layerName = 'res5c_relu';

elseif netName == "mobilenetv2"

layerName = 'out_relu';

end

end

%% CAMshow function

% Please note, this function was provided by the Mathworks example

function CAMshow(im,CAM)

imSize = size(im);

CAM = imresize(CAM,imSize(1:2));

CAM = normalizeImage(CAM);

CAM(CAM<0.2) = 0;

cmap = jet(255).*linspace(0,1,255)';

CAM = ind2rgb(uint8(CAM*255),cmap)*255;

combinedImage = double(rgb2gray(im))/2 + CAM;

combinedImage = normalizeImage(combinedImage)*255;

imshow(uint8(combinedImage));

end
