# Supplementary material for: Deep learning-based classification of the capillary ultrastructure in human skeletal muscles
Source: Front Mol Biosci. 2024 May 1;11:1363384. doi: 10.3389/fmolb.2024.1363384 (PMC11094256; doi:10.3389/fmolb.2024.1363384)
Supplement: Supplementary file 3 [file Image1.pdf]

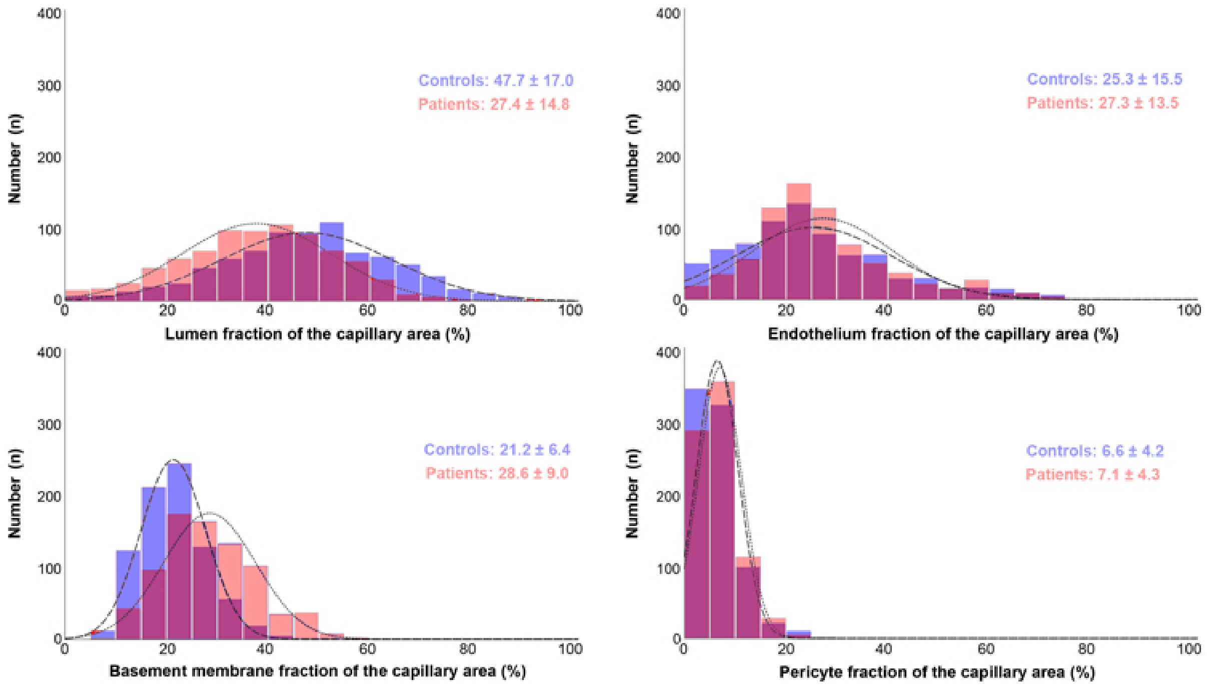

Supplemental figure 1: Histograms representing the frequency distribution of the compartment fractions related to total the capillary area in human skeletal muscle grouped by the absence or presence of systemic pathologies. The morphometric values for the capillary structure of 879 electron micrographs from control participants and 836 electron micrographs from patients were taken from the original studies listed in Materials/Methods. They structural indicators were determined using tablet-based image analysis and represent mean ± standard deviation.

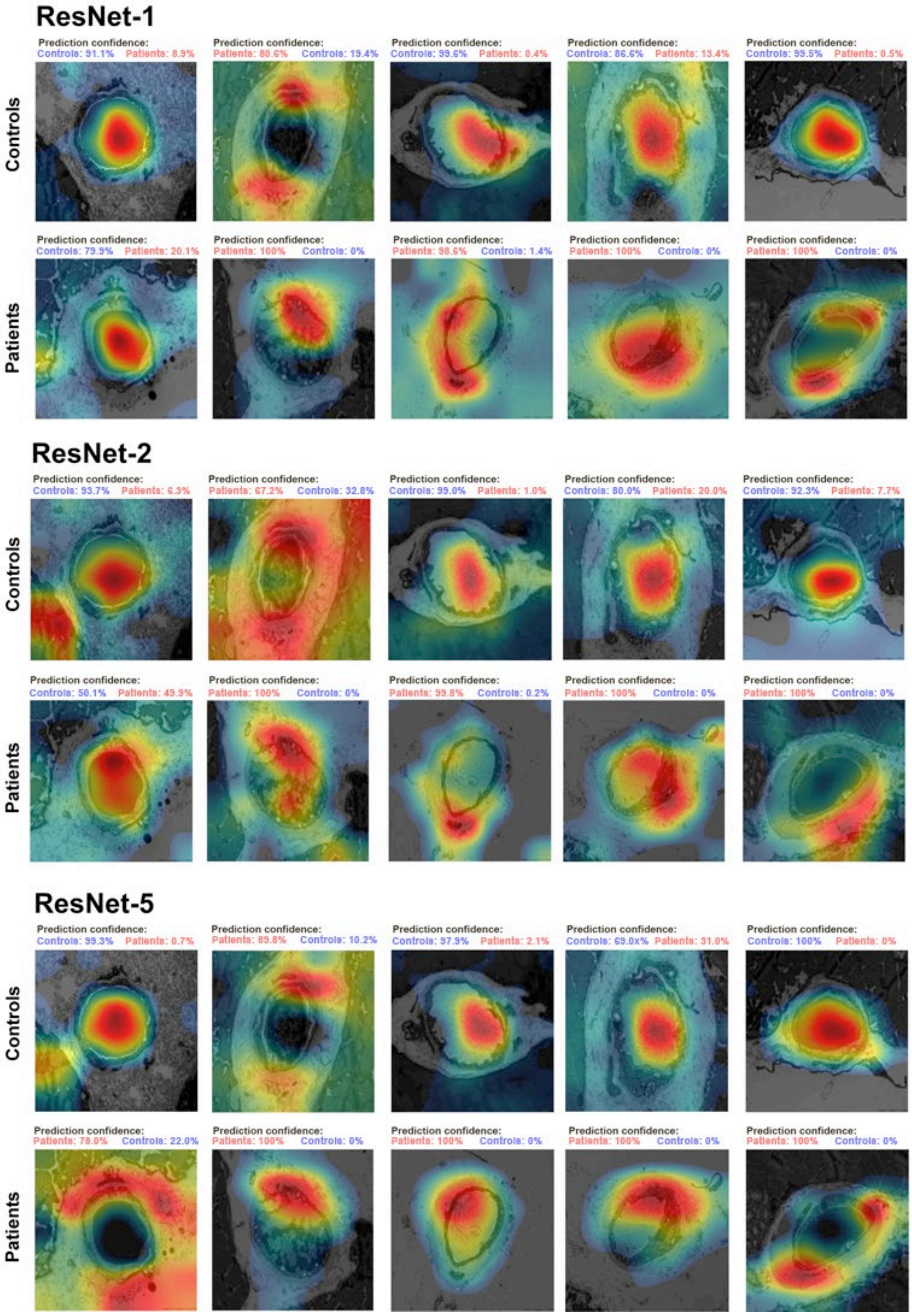

Supplemental figure 2: Visualization of network activation patterns of ResNet1, ResNet2 and ResNet5. Networks performing above the arbitrary threshold of 60% classification accuracy on the validation data during the training process were stored for in-depth evaluation. Based on the results of this evaluation, the best three networks were further evaluated with class activation mapping (CAM), i.e., parametric visualization of their activation patterns to find the morphology responsible for the network prediction. Shown are the class activation heat maps of representative electron micrographs from capillaries of controls (n=5) and patients (n=5). Red represents the highest activation level for a CNN. Note that the areas representing pericyte debridement appear to be the areas of highest activation. Further, please note the incorrect predictions (i.e. picture 2 from the left side in the control section for all CNN as well as picture 1 from the left side in the patient section for ResNet1 and ResNet2).
